# Immunomodulation by an Omega-6 Fatty Acid Reduced Mixed Lipid Emulsion in Murine Acute Respiratory Distress Syndrome

**DOI:** 10.3390/jcm9072048

**Published:** 2020-06-29

**Authors:** Matthias Hecker, Matthias Rose, Andreas Hecker, Hartmut Dietrich, Martina B. Schaefer, Natascha Sommer, Werner Seeger, Konstantin Mayer

**Affiliations:** 1 University of Giessen and Marburg Lung Center (UGMLC), University Hospital of Giessen, Justus-Liebig-University of Giessen, 35392 Giessen, Germany; Matthias.Rose@innere.med.uni-giessen.de (M.R.); Hartmut.Dietrich@innere.med.uni-giessen.de (H.D.); Martina.Schaefer@innere.med.uni-giessen.de (M.B.S.); Natascha.Sommer@innere.med.uni-giessen.de (N.S.); Werner.Seeger@innere.med.uni-giessen.de (W.S.); Konstantin.Mayer@innere.med.uni-giessen.de (K.M.); 2 Department of General and Thoracic Surgery, University Hospital of Giessen, Justus-Liebig-University of Giessen, 35392 Giessen, Germany; Andreas.Hecker@chiru.med.uni-giessen.de

**Keywords:** olive oil, acute respiratory distress syndrome, lipid emulsion, inflammation, fish

## Abstract

Background: Acute respiratory distress syndrome (ARDS) is associated with both high morbidity and mortality in intensive care units worldwide. Patients with ARDS often require parenteral nutrition with lipid emulsions as essential components. In the present study, we assessed the immunomodulatory and apoptotic effects of a modern, n-6-reduced lipid emulsion mixture in murine ARDS. Methods: Mice received an infusion of either normal saline solution, pure long-chain triglyceride (LCT) emulsion, or SMOF (soybean oil, medium-chain triglycerides, olive oil, and fish oil) before a lipopolysaccharide (LPS) challenge. Mice were sacrificed at different time points (0, 24, or 72 h) after ARDS induction, and an analysis of inflammatory cytokines, protein concentrations, and the cellular composition of the alveolar and interstitial compartments was performed with special focus on alveolar apoptosis and necrosis. Results: Mice infused with SMOF showed decreased leukocyte invasion, protein leakage, myeloperoxidase activity, and cytokine production in alveolar spaces after LPS challenge compared to animals that received LCT. There were fewer cells in the lung interstitium of the SMOF group compared to the LCT group. Both lipid emulsions exerted pro-apoptotic and pro-necrotic properties on alveolar immune cells, with significantly increased necrosis in mice infused with LCT compared to SMOF. Conclusion: SMOF has both anti-inflammatory and pro-resolving influences in murine ARDS. Partial replacement of n-6 fatty acids with n-3/n-9 fatty acids may therefore benefit critically ill patients at risk for ARDS who require parenteral nutrition.

## 1. Introduction

Acute respiratory distress syndrome (ARDS) is a common disorder associated with high morbidity and mortality in intensive care units worldwide [1]. Pathophysiologically, ARDS is characterized by initial alveolar epithelial and endothelial injuries that lead to the progressive development of non-cardiogenic protein-rich pulmonary edema, refractory hypoxemia, and finally to respiratory failure [2]. Furthermore, the disruption of the alveolar–capillary barrier enables the invasion of neutrophils into the interstitial and alveolar spaces with subsequent formation of pro-inflammatory mediators such as cytokines and eicosanoids [3,4]. Despite decades of intensive research on this disease, at present, no proven pharmaceutical option is available for ARDS patients, and this is reflected by an unacceptably high mortality rate of 30–40%, mostly due to severe sepsis [5].

Lipid emulsions (LEs) are regarded as essential components of parenteral nutrition regimens applied clinically to critically ill patients [6]. In addition to providing adequate caloric support, both experimental and clinical studies have indicated that LEs might exert immunomodulatory properties [7,8,9]. In this context, LEs have been shown to modify and influence cytokine release, leukocyte function, and the generation of lipid mediators which display both pro- and anti-inflammatory properties [10,11,12]. The clinical impact of these immunological properties on intensive care patients is the subject of increasing research interest. Furthermore, we and others have shown that LEs are also capable of inducing apoptosis in immune cells, findings that may impact the influence of LEs on immune responses under certain circumstances [13,14,15,16]. Historically, fatty-acid supplementation exclusively relied on soybean- (SO) or safflower-oil-based long-chain triglycerides (LCT) largely made up of linolenic acid, an n-6 polyunsaturated fatty acid that serves as a precursor for arachidonic acid. Numerous clinical, in-vivo, and in-vitro studies of this LE reported both immunosuppressive effects and increased generation of pro-inflammatory n-6-derived lipid mediators [17,18]. Therefore, to reduce SO content, pharmaceutical companies developed a variety of LEs containing medium-chain triglycerides (MCTs), fish oil (FO), and/or olive oil (OO) in addition to SO formulations. Regarding immunological effects, FO-containing LEs provide n-3 polyunsaturated fatty acids that may provide anti-inflammatory modulation, but the few studies of parenterally applied FO-containing LEs in ARDS patients have shown inconsistent results [11]. Even fewer data were available for parenterally applied OO-based LEs in this patient cohort, but the rationale to include OO (with oleic acid as a major component) was the potential for reducing lipid peroxidation and the generation of reactive oxygen species.

For the first time, the present study investigated the effects of conventional SO-based LEs in a murine model of endotoxin-induced acute respiratory distress syndrome (ARDS) compared to the effects of a third-generation formulation: SMOF (soybean oil (30%), medium-chain triglycerides (30%), olive oil (25%), and fish oil (15%) supplemented with the antioxidant α-tocopherol). We made use of continuous, long-term lipid infusion followed by an endotoxin challenge. Subsequent in-vivo and in-vitro analyses focused on the spatial distribution of relevant immune cells in different lung compartments and analyses of pro-apoptotic properties.

## 2. Materials and Methods

### 2.1. Reagents

An SO-based emulsion (LCT; Lipoven 20%^®^) and an OO-based emulsion (SMOF; SMOFlipid 20%^®^) were obtained from Fresenius-Kabi (Bad Homburg, Germany). The fatty acid compositions of both lipid emulsions are listed in Table 1. All reagent-grade chemicals were obtained from Merck (Darmstadt, Germany). Lipopolysaccharide (LPS, O111:B4) from Escherichia coli was obtained from Sigma-Aldrich (Dreisenhofen, Germany).

### 2.2. Animals and Experimental Protocol

Local government authorities and university officials responsible for animal protection approved the study (GI20/10-120/2014). BALB/c mice were housed using a 12 h day/12 h night cycle of lighting under pathogen-free conditions. Animals 13–15 weeks old (22–24 g weight) were used for the experiments. For each mouse, the implantation of a jugular-vein catheter and adaptation to an osmotic mini-pump (Alzet, Cupertino, CA) were performed as described previously [19].

Seven days after the implantation of a central venous catheter, the original osmotic pump was exchanged. Pumps delivered either 200 µL per day of LCT, SMOF or normal saline (NaCl) over three days, with mice allowed access to water and chow ad libitum. The amount of lipid infused was equivalent to 1.5 g/kg/day, but, given that the energy expenditure in mice is approximately three times higher compared to in humans, the lipid infusions were close to the lower limit of recommended lipids for human parenteral nutrition. During infusions, the mice also received low-dose unfractionated heparin injected subcutaneously.

### 2.3. LPS-induced Acute Lung Injury in the Murine Model

After completion of the infusion regimen, each mouse was anesthetized with xylazine/ketamine, a small catheter was inserted in its trachea, and LPS (0 or 10 µg in 200 µL normal saline) was administered using a Microsprayer (Penn-Century Inc., Philadelphia, PA, USA). Four, twenty-four, or forty-eight hours after LPS administration, mice were re-anesthetized as described before, and volumetric computer tomography of the lung was performed. After these volumetric measurements, mice were sacrificed by anesthetic overdose, and the lungs were either harvested for further histological examinations or a bronchoalveolar lavage (BAL) was performed as previously described [19].

### 2.4. Assessment of Lung Edema

The degree of lung edema was estimated by protein analysis of the bronchoalveolar lavage according to the method of Lowry [20].

### 2.5. BAL Leukocyte Counts

Mice were killed by anesthetic overdose, and BAL was performed in situ as described [19]. Alveolar-recruited leukocytes recovered from the lungs of both LPS-challenged and control mice were counted using a counting chamber positioned under a light microscope. The differential leukocyte counting-analysis was performed after hematoxylin and eosin staining.

### 2.6. Enzyme-linked Immunosorbent Assay (ELISA)

ELISA kits for tumor-necrosis factor (TNF)-α, macrophage inflammatory protein (MIP)-2, prostaglandin (PG) E2 (all obtained from R&D Systems, Wiesbaden, Germany), and thomboxane (Tx) B2 (Assay Designs, Ann Arbor, Michigan) were used to determine levels in BAL samples according to the manufacturers’ instructions.

### 2.7. Myeloperoxidase Assay

Lung myeloperoxidase (MPO) was determined as an indicator of tissue neutrophil accumulation after LPS challenge, as previously described. Lung samples were stored at −80 °C. After weighing, lung samples were homogenized, sonicated, and centrifuged at 25,000× *g*. MPO activity was calculated from changes in absorbance (460 nm) resulting from the decomposition of H_2_O_2_ in the presence of o-dianisidine.

### 2.8. Assessment of Apoptosis by Flow Cytometry

Alveolar cells were gently minced to dissociate cells in RPMI1640 medium containing 5% FCS. After several washing steps, cells were resuspended in 50 µL binding buffer (10 mM HEPES, 140 mM NaCl, 2.5 mM CaCl_2_, pH 7.4) containing FITC-conjugated annexin V and incubated for 15 min before adding 7-AAD. Leukocyte sub-populations were identified based on characteristic forward- and side-scatter properties. The percentages of apoptotic cells were detected by annexin V staining, and necrotic cells were excluded by 7-AAD labeling. All experiments were performed on a FACScan according to the manufacturer’s instructions (BD Biosciences, Franklin Lakes, NY, USA).

### 2.9. Flow Cytometry and Staining Procedure

Cellular phenotyping was performed on a FACScan flow cytometer (Becton Dickinson, San Jose, CA, USA). All antibodies were purchased from BD Pharmingen (Germany). Isotype-matched control antibodies were ordered form the same company. In brief, after performing an automated cell count, blood was incubated with erythocyte lysing buffer (BD-Pharm Lyse, BD Biosciences, Germany) at room temperature and centrifuged, and supernatant was subsequently discarded. Surface antibody or isotype staining time was 30 min followed by washing with staining buffer (1 × PBS/5% FBC, both reagents from PAA, Germany).

### 2.10. Statistics

Data are presented as the means ±SEMs. Independent experiments (n = 6–8) were performed by group and by time point. The D’Agostino–Pearson omnibus normality test was used to assess the normal distribution of the variables. A two-way analysis of variance (ANOVA) was performed to test for differences between different infusion groups. Post-hoc analyses were carried out using Student–Newman–Keuls tests. Probability (*p*) values < 0.05 were considered statistically significant. Analyses were carried out using SigmaStat^®^ 3.5 software for Windows (Version 3.5, Systat Software Inc., San Jose, CA, USA. 2007).

## 3. Results

### 3.1. Protein Concentrations of BAL Fluids

**Figure 1:**
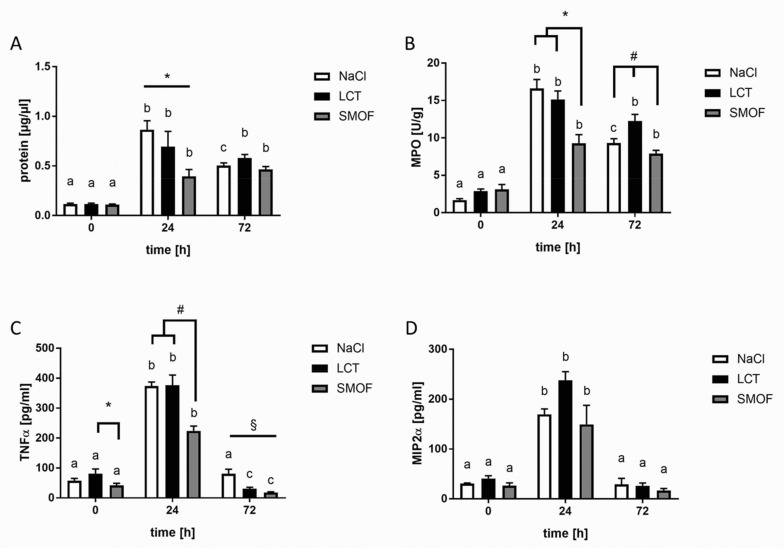
In a murine ARDS model, (**A**) impact of lipid emulsions on alveolar edema formation, (**B**) tissue neutrophil accumulation, and (**C**,**D**) cytokine generation. A: 24 h after LPS challenge, mice in the SMOF group displayed significantly reduced edema formation compared to the NaCl and LCT groups (*), *p* < 0.01). B: The lowest MPO activities were measured in the SMOF group 24 h after ARDS induction compared to the LCT and NaCl groups (*, *p* < 0.01). MPO activity 72 h after LPS administration was highest in lung tissue from mice receiving LCT compared to the SMOF and NaCl groups (#, *p* < 0.01). **C**: TNF-α concentrations in BAL fluid from mice infused with SMOF were significantly lower compared to concentrations from LCT mice (*, *p* < 0.01). After 24 h, TNF-α concentrations in the SMOF group were the lowest compared to NaCl and LCT (#, *p* < 0.01). The highest TNF-α values were measured in the NaCl group after 72 h compared to LCT and SMOF (§, *p* < 0.01). **D**: All infusion groups showed no significant differences concerning MIP-2 concentration.

First, we assessed protein extravasation in BAL samples as a marker for edema formation and barrier integrity. Basal protein levels were comparable among all unstimulated groups. For all mouse groups (NaCl, LCT, and SMOF), protein concentrations in BAL samples increased significantly 24 h after LPS stimulation (a vs. b, *p* < 0.01) and declined after 72 h, but values were still significantly elevated compared to unstimulated controls (a vs. b, *p* < 0.01). Only in the NaCl group did the 24-h protein concentration differ significantly from the 72-h concentration (b vs. c, *p* < 0.01). As shown in Figure 1A, 24 h after LPS challenge, mice in the SMOF group displayed significantly reduced protein concentrations compared to the NaCl and LCT groups (*, *p* < 0.01), whereas, after 72 h, all infusion group BAL samples were determined to have similar protein levels.

### 3.2. Accumulation of Neutrophils in Lung Tissue

To assess neutrophil accumulation in the lungs, MPO activity was determined before and 24 h and 72 h after LPS challenge in all groups. Without LPS stimulation, MPO activity did not differ among the infusion groups. Administration of 10 µg of LPS induced a significant increase in MPO activity after 24 h in all groups and declined after 72 h, but this latter activity was still significantly different from untreated controls (see Figure 1B; a vs. b, *p* < 0.01). Only in the SMOF group did the 24-h and 72-h values not differ significantly (c, n.s.). Comparisons of MPO activities in the different infusion regimes 24 h after ARDS induction revealed the lowest activity in the SMOF group compared to the LCT and NaCl groups (*, *p* < 0.01). MPO activity 72 h after LPS administration was highest in lung tissue from mice receiving LCT compared to the SMOF and NaCl groups (#, *p* < 0.01).

### 3.3. Generation of Cytokines in ARDS

As depicted in Figure 1C, the TNF-α concentration in BAL fluid differed significantly for animals treated with LCT or SMOF at all time points (a vs. b vs. c, *p* < 0.01), whereas, in the NaCl cohort, only the 24 h value after LPS challenge was different from the other two times (a vs. b vs. a, *p* < 0.01). Comparing the different LEs under unstimulated conditions, the TNF-α concentrations in BAL fluid from mice infused with SMOF were significantly lower compared to concentrations from LCT mice (*, *p* < 0.01). BAL concentrations of TNF-α increased significantly 24 h after LPS challenge and significantly declined 72 h after stimulation. SMOF-infused mice displayed significantly lower TNF-α values after 24 h compared to both unstimulated controls and the LCT group (#, *p* < 0.01). At 72 h after LPS stimulation, the NaCl group displayed the highest BAL TNF-α concentrations compared to the LCT and SMOF groups (§, *p* < 0.01).

Next, we focused on MIP-2 as another example of a pro-inflammatory cytokine. For all the infusion groups, MIP-2 concentrations were significantly increased 24 h after LPS administration (a vs. b, *p* < 0.01). MIP-2 levels in BAL samples significantly decreased after 72 h compared to 24 h in all groups. For all infusion groups, no significant differences were observed between the time points tested (Figure 1D).

For all figures: differences concerning the time course (0h, 24 h, 72 h) for the respective group (NaCl, LCT, SMOF) are significant if the letters (a, b, c) are different (*p* < 0.01). Data are given as mean ±SEM (*n* = 6–8 independent experiments each).

### 3.4. Effect of Lipid Emulsions on Alveolar Recruitment of Leukocytes in LPS-induced Acute Respiratory Distress Syndrome

**Figure 2:**
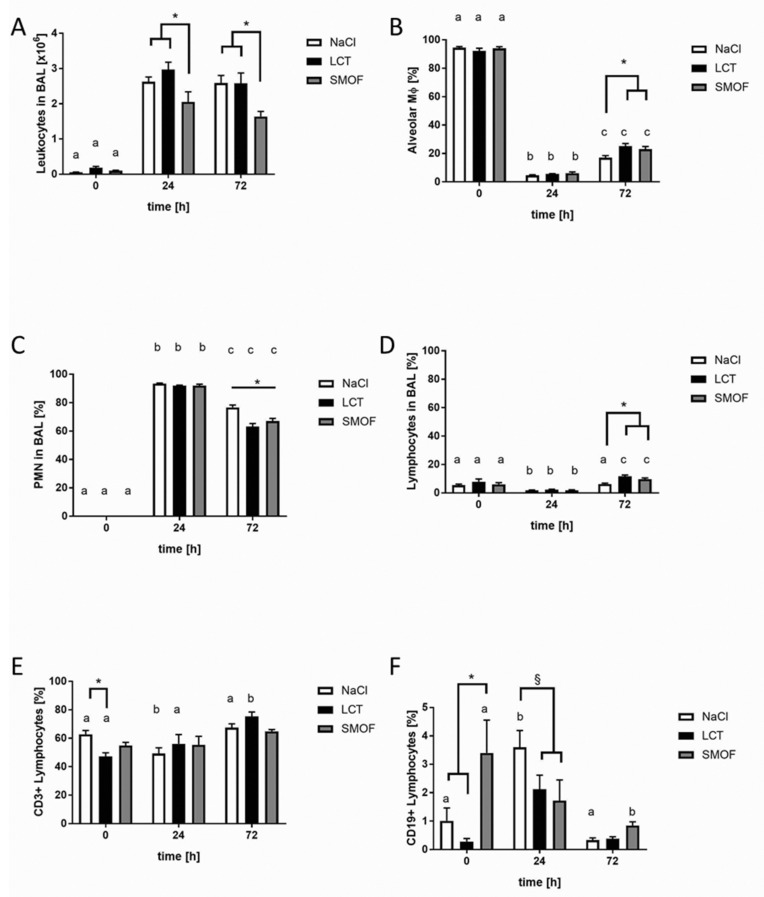
Effect of lipid emulsions on alveolar recruitment of leukocyte subtypes in LPS-induced acute respiratory distress syndrome. (**A**): At both 24 h and 72 h after induction of ARDS, the SMOF mouse group showed the lowest number of alveolar leukocytes compared to the NaCl and LCT groups (*, *p* < 0.01). (**B**): After 72 h of LPS stimulation, the NaCl group displayed the lowest fraction of alveolar Mϕ-type cells compared to animals receiving LEs (*, *p* < 0.01). (**C**): After 72 h, mice receiving NaCl showed the highest BAL content of PMNs compared to other groups (*, *p* < 0.01). (**D**): The NaCl group showed significantly reduced lymphocytes compared to the other groups 72 h after LPS instillation (*, *p* < 0.01). (**E**): In unstimulated mice, CD3+ lymphocytes were reduced in the LCT group compared to NaCl (*, *p* < 0.01). (**F**): After 24 h, the highest proportion of CD19+ lymphocytes was in NaCl mice compared to LCT and SMOF mice (§, *p* < 0.01).

Next, we sought to analyze the effect of the different LEs on alveolar recruitment of leukocytes and their relevant sub-populations, as shown in Figure 2. After LPS stimulation, leukocytes migrated into the alveolar space, and their numbers in BAL fluid increased significantly after 24 h and 72 h in all groups compared to unstimulated controls (a, *p* < 0.01). At both 24 h and 72 h after induction of ARDS, the SMOF mouse group showed the lowest number of alveolar leukocytes compared to the NaCl and LCT groups (*, *p* < 0.01, Figure 2A). A differential cell-count analysis of BAL leukocytes was used to elucidate the percentage of monocytes/macrophages (Mϕ), polymononuclear cells (PMNs), and lymphocytes (sub-divided into CD3+- and CD19+-groups). As expected, LPS induced a significant decline in alveolar Mϕ-type cells 24 h after administration compared to unstimulated controls for all groups (a vs. b, *p* < 0.01; Figure 2B). After 72 h, alveolar Mϕ-type cells increased significantly compared to the 24 h values of all the intervention groups (b vs. c, *p* < 0.01). After 72 h of LPS stimulation, the NaCl group displayed the lowest fraction of alveolar Mϕ-type cells compared to animals receiving LEs (*, *p* < 0.01, Figure 2B). The percentage of PMNs in BAL fluid increased significantly both 24 h and 72 h after induction of ARDS (Figure 2C; a vs. b vs. c, *p* < 0.01). After 72 h, mice that received NaCl showed the highest BAL content of PMNs compared to other groups (*, *p* < 0.01, Figure 2C). For lymphocytes, the lowest percentage was measured 24 h after LPS administration compared to the other time points (b, *p* < 0.01, Figure 2D). Animals infused with NaCl also showed significantly reduced lymphocytes compared to the LE groups 72h after LPS instillation (*, *p* < 0.01, Figure 2D). Sub-analysis of the lymphocyte population indicated that, under unstimulated conditions, application of LCT significantly reduced CD3+ lymphocytes compared to the NaCl group (*, *p* < 0.01, Figure 2E). For CD19+ lymphocytes, SMOF infusion significantly elevated the proportion of these cells compared to the other groups before the LPS challenge (*, *p* < 0.01, Figure 2F). After 24 h, the highest proportion was in NaCl mice compared to LCT and SMOF mice (§, *p* < 0.01, Figure 2F).

For all figures, differences concerning the time course (0h, 24 h, 72 h) for the respective group (NaCl, LCT, SMOF) are significant if the letters (a, b, c) are different (*p* < 0.01). Data are given as mean ±SEM (*n* = 6–8 independent experiments each).

### 3.5. Assessment of Apoptosis in LPS-induced Acute Respiratory Distress Syndrome

**Figure 3:**
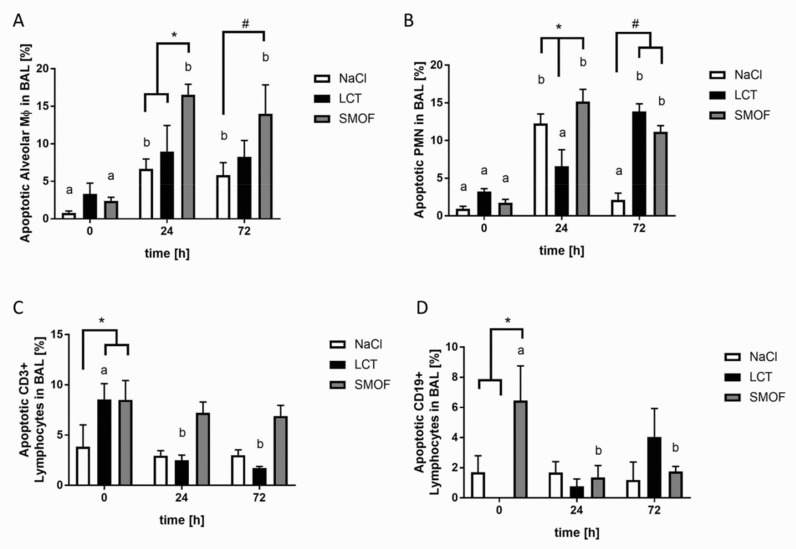
Assessment of alveolar cell apoptosis in LPS-induced acute respiratory distress syndrome. (**A**): Mice receiving SMOF displayed significantly increased apoptotic alveolar Mϕ-type cells compared to NaCl and LCT mice after 24 h (*, *p* < 0.01). After 72 h, the percentage of apoptotic alveolar Mϕ-type cells was higher in the SMOF group compared to the NaCl group (#, *p* < 0.01). (**B**): For PMNs in BAL fluid, all groups significantly differed from each other 24 h after LPS challenge (*, *p* < 0.01). After 72 h, the NaCl group showed significantly fewer apoptotic PMNs compared to the LCT and SMOF groups (#, *p* < 0.01).(**C**): Mice infused with NaCl had a lower percentage of apoptotic CD3+ lymphocytes in BAL fluid compared to LCT and SMOF mice under unstimulated conditions (*, *p* < 0.01). (**D**): Without LPS stimulation, SMOF application induced significantly higher apoptosis in this cell type compared to the NaCl and LCT groups (*, *p* < 0.01). For all figures, differences concerning the time course (0 h, 24 h, 72 h) for the respective group (NaCl, LCT, SMOF) are significant if the letters (a, b, c) are different (*p* < 0.01). Data are given as mean ± SEM (*n* = 6–8 independent experiments each).

Apoptosis was assessed in different alveolar cell types within the context of murine ARDS (Figure 3). At both 24 h and 72 h after ARDS induction, mice in the NaCl and SMOF groups showed increased apoptotic alveolar Mϕ-type cells compared to unstimulated controls (a vs. b, *p* < 0.01, Figure 3A). Mice that received SMOF displayed significantly more apoptotic cells compared to NaCl and LCT mice after 24 h (*, *p* < 0.01, Figure 3A). After 72 h, the percentage of apoptotic alveolar Mϕ-type cells was higher in the SMOF group compared to the NaCl group (#, *p* < 0.01, Figure 3A). For PMNs in BAL fluid, all groups significantly differed from each other 24 h after LPS challenge (*, *p* < 0.01, Figure 3B). After 72 h, the NaCl group showed significantly fewer apoptotic PMNs compared to the LCT and SMOF groups (#, *p* < 0.01, Figure 3B). As depicted in Figure 3C, mice infused with NaCl had a lower percentage of apoptotic CD3+ lymphocytes in BAL fluid compared to LCT and SMOF mice under unstimulated conditions (*, *p* < 0.01, Figure 3C). Furthermore, mice that received LCT had the highest percentage of apoptotic CD3+ lymphocytes under unstimulated conditions compared to the other time points (a vs. b, *p* < 0.01). Similar results were obtained for apoptotic CD19+ lymphocytes in BAL fluid (Figure 3D). Without LPS stimulation, SMOF application induced significantly higher apoptosis in this cell type compared to the NaCl and LCT groups (*, *p* < 0.01, Figure 3D). In addition, counts of apoptotic CD19+ lymphocytes in SMOF-cohort BAL fluid were highest under unstimulated conditions compared to the other time points (a vs. b, *p* < 0.01).

### 3.6. Analysis of Necrosis in LPS-induced Acute Respiratory Distress Syndrome

**Figure 4:**
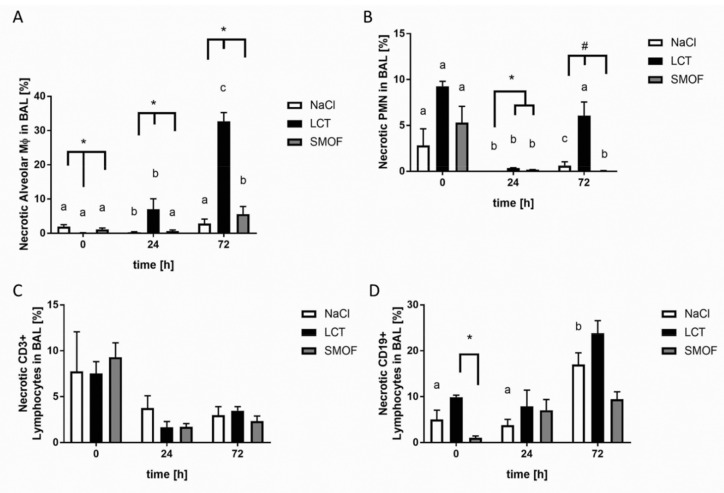
Analysis of necrosis in LPS-induced acute respiratory distress syndrome. (**A**): Assessment of necrotic alveolar Mϕ-type cells revealed that mice that received LCT infusions significantly differed from the other groups (*, *p* < 0.01). (**B**): For necrotic PMNs, mice that received either LCT or SMOF had significantly higher proportions of necrotic PMNs compared to the NaCl group 24 h after LPS stimulation (*, *p* < 0.01). (**C**): No significant changes in necrotic CD3+ lymphocytes were found. (**D**): For necrotic CD19+ lymphocytes in BAL fluid, mice infused with LCT displayed significantly more necrosis compared to SMOF-infused mice under non-inflammatory conditions (*, *p* < 0.01). For all figures, differences concerning the time course (0 h, 24 h, 72 h) for the respective group (NaCl, LCT, SMOF) are significant if the letters (a, b, c) are different (*p* < 0.01). Data are given as mean ± SEM (*n* = 6–8 independent experiments each).

Assessment of necrotic alveolar Mϕ-type cells revealed that mice that received LCT infusions significantly differed from the other groups (*, *p* < 0.01, Figure 4A). The highest LCT group values were seen 24 h and 72 h after ARDS induction compared to unstimulated controls (a vs. b vs. c; *p* < 0.01). For necrotic PMNs, mice that received either LCT or SMOF had significantly higher proportions of necrotic PMNs compared to the NaCl group 24 h after LPS stimulation (*, *p* < 0.01, Figure 4B). After 72 h of stimulation, all groups differed significantly from each other, with the highest value seen in the LCT group (#, *p* < 0.01, Figure 4B). No significant changes in necrotic CD3+ lymphocytes were found (Figure 4C). For necrotic CD19+ lymphocytes in BAL fluid, mice infused with LCT displayed significantly more necrosis compared to SMOF-infused mice under non-inflammatory conditions (*, *p* < 0.01, Figure 4D).

### 3.7. Effect of Lipid Emulsions on Lung-tissue Leukocytes in LPS-induced Acute Respiratory Distress Syndrome

**Figure 5:**
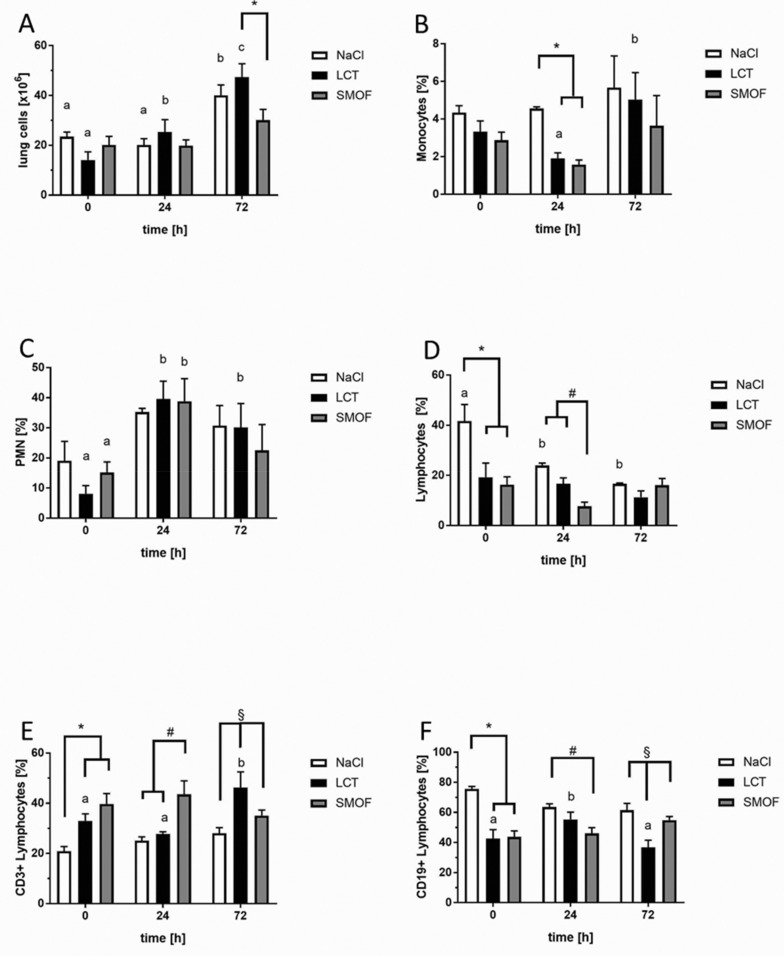
Effect of lipid emulsions on lung-tissue leukocytes in LPS-induced acute respiratory distress syndrome. (**A**): Interstitial cells were significantly reduced 72 h after induction of ARDS in animals receiving SMOF compared to the LCT group (*, *p* < 0.01). (**B**): Analysis of the leukocyte sub-populations revealed a significant decline in the percentage of monocytes in the LCT and SMOF groups compared to the NaCl group 24 h after ARDS induction (*, *p* < 0.01). (**C**): For PMNs, no significant differences among the different infusion groups were detected. (**D**): For interstitial lymphocytes, a significantly higher percentage was seen in NaCl-group mice without LPS stimulation compared to the LCT and SMOF groups (*, *p* < 0.01). At 24 h after LPS administration, the percentage of lymphocytes was lowest in the SMOF group (#, *p* < 0.01). (**E**): Without stimulation, mice that received NaCl infusions had the lowest percentage of CD3+ lymphocytes compared to the LCT and SMOF groups (*, *p* < 0.01). After 24 h, the highest value for CD3+ lymphocytes was in the SMOF group (#, *p* < 0.01), whereas, after 72 h, the LCT group differed significantly from the other infusion groups (§, *p* < 0.01). (**F**): Interstitial CD19+ lymphocytes were significantly increased in the NaCl group before LPS stimulation (*, *p* < 0.01). Mice that received SMOF displayed a significant reduction in cells compared to the NaCl group 24 h after ARDS induction (#, *p* < 0.01). After 72 h, the lowest interstitial proportion of CD19+ lymphocytes was seen in the LCT group compared to the SMOF and NaCl groups (§, *p* < 0.01). For all figures, differences concerning the time course (0 h, 24 h, 72 h) for the respective group (NaCl, LCT, SMOF) are significant if the letters (a, b, c) are different (*p* < 0.01). Data are given as mean ±SEM (*n* = 6–8 independent experiments each).

Lastly, the cellular composition within lung interstitium in response to LPS stimulation and infusion of LEs was assessed (Figure 5). Interstitial cells were significantly reduced 72 h after induction of ARDS in animals receiving SMOF compared to the LCT group (*, *p* < 0.01, Figure 5A). For the NaCl group, the highest interstitial cell counts were detected 72 h after LPS administration compared to the other time points (a vs. b, *p* < 0.01). For LCT, the cell counts differed significantly among all time points (a vs. b vs. c, *p* < 0.01). Analysis of the leukocyte sub-populations revealed a significant decline in the percentage of monocytes in the LCT and SMOF groups compared to the NaCl group 24 h after ARDS induction (*, *p* < 0.01, Figure 5B). For PMNs, no significant differences among the different infusion groups were detected (Figure 5C). For interstitial lymphocytes, a significantly higher percentage was seen in NaCl-group mice without LPS stimulation compared to the LCT and SMOF groups (*, *p* < 0.01, Figure 5D). At 24 h after LPS administration, the percentage of lymphocytes was lowest in the SMOF group (#, *p* < 0.01, Figure 5D). Lymphocyte differentiation analysis showed that, before LPS challenge, mice that received NaCl infusions had the lowest percentage of CD3+ lymphocytes compared to the LCT and SMOF groups (*, *p* < 0.01, Figure 5E). After 24 h, the highest value for CD3+ lymphocytes was in the SMOF group (#, *p* < 0.01, Figure 5E), whereas, after 72 h, the LCT group differed significantly from the other infusion groups (§, *p* < 0.01, Figure 5E). Analysis of interstitial CD19+ lymphocytes revealed a significant increase in the NaCl group before LPS stimulation (*, *p* < 0.01, Figure 5F). Mice that received SMOF displayed a significant reduction in cells compared to the NaCl group 24 h after ARDS induction (#, *p* < 0.01, Figure 5F). After 72 h, the lowest interstitial proportion of CD19+ lymphocytes was seen in the LCT group compared to the SMOF and NaCl groups (§, *p* < 0.01, Figure 5F).

## 4. Discussion

In the present study, we investigated the impact of an n-6-reduced LE mixture, SMOF, compared to a conventional, pure, LCT emulsion in a murine model of ARDS. Experiments revealed that mice treated with SMOF displayed reduced invasions of leukocytes in both alveolar and tissue compartments. In addition, protein leakage and the generation of pro-inflammatory cytokines were both lowered compared to animals infused with LCT. Importantly, both LEs significantly induced apoptosis in alveolar Mϕ-type cells and PMNs upon LPS stimulation. Furthermore, the rate of necrosis was significantly elevated in these cell types after LCT infusion.

The present report’s major finding is the immunomodulatory impact of SMOF on the development and progression of murine ARDS. SMOF was introduced commercially as a so-called fourth-generation LE composed of soybean oil, medium chain triglycerides, olive oil, and fish oil. In addition to providing sufficient energy and essential fatty acids, the rationale for choosing this composition was to decrease the proportion of n-6 polyunsaturated fatty acids by the addition of both the very-long-chain n-3 fatty acids eicosapentaenoic acid (EPA) and docosahexaenoic acid (DHA) and monounsaturated oleic acid. To date, several biological mechanisms have been identified that explain the beneficial effects of LEs containing reduced n-6 content and supplemented with n-3 fatty acids: partial replacement of arachadonic acid among cell-membrane phospholipids by n-3 fatty acids, EPA and DHA [21]. These alterations have a direct impact on eicosanoid synthesis and membrane composition, and subsequently change a cell’s signal-transduction machinery [22,23]. In support of this, Camandola and colleagues were able to show that n-6 fatty acids, unlike EPA, stimulate nuclear translocation and subsequent activation of the transcription factor NF-κB, a key mediator of inflammatory processes [24]. These pathomechanisms may explain the present findings of reduced levels of TNF-α and MIP-2 in the BAL fluid of SMOF-treated mice compared to animals treated with LCT.

Furthermore, our experiments showed reduced invasion of leukocytes into both alveolar and tissue compartments in mice treated with SMOF compared to those infused with LCT. The invasion of neutrophils into the alveolar compartment is regarded as a characteristic feature of ARDS and is also a well-documented response in both mice and humans after LPS challenge [11,19]. The process of neutrophils transmigrating though the endothelial–epithelial barrier after LPS stimulation is complex and only partly understood. n-3 fatty acids may influence and modify this multistep cascade via reduced presentation of endothelial adhesion molecules [25] and the attenuation of monocyte rolling [26]. In this context, n-3-induced changes in lipid-dependent signaling pathways (so-called lipid signaling), such as the generation of inositol phosphates and the formation of lipid rafts, may also be relevant [6,27]. Furthermore, the recent discovery of resolvins as a novel class of fish-oil-derived lipid mediators is an emerging field of research investigating not only their anti-inflammatory properties but also the inflammation-resolving effects of fish oil [28,29,30,31].

Unlike the effects of n-6 and n-3 fatty acids, little is known about the immunological role of the n-9 fatty acid oleic acid as a main component in olive oil (another part of SMOF) in ARDS/severe inflammation. Oleic acid is a monounsaturated omega-9 fatty acid abbreviated with a lipid number of 18:1 *cis*-9. Most studies thus far have used olive-oil-based LEs such as ClinOleic^®^ (an 80:20 mixture of olive and soybean oil). One rationale for the use of olive oil is because of its high monounsaturated fatty acid content and anti-oxidative effects, especially in cardiovascular disorders [32]. For the impact of olive-oil based LEs on the immune system, Reimund and colleagues described their immunological properties as approximately neutral using an in-vitro assay compared to LCT [33]. In line with this finding, Granato et al. reported that ClinOleic^®^ does not affect lymphocyte proliferation in vitro [34], and Buenestado and colleagues demonstrated that ClinOleic^®^ showed lower in-vitro and in-vivo impact on neutrophil function compared to both LCT and LCT–MCT [35]. In addition to these basic science experiments, clinical trials investigating olive-oil-based LEs in critically ill patients [36], those with severe burns [37], and those needing home parenteral nutrition [38] demonstrated only minor effects on immune and inflammatory responses.

The present study was performed using SMOF, a fourth-generation LE. To the best of our knowledge, this LE has not yet been investigated in (experimental) ARDS or in inflammation experiments. The rationale for the development of these novel n-6-reduced LE mixtures was to avoid the excessive intake of pro-inflammatory n-6 fatty acids, mainly by optimizing the n-6/n-3 ratio and including olive oil as the source of monounsaturated fatty acid. Currently, there are few reports of clinical SMOF trials in the literature. In addition to early studies on safety and efficacy, more recent clinical work has examined SMOF in pediatrics and neonatology [39,40,41,42], demonstrating that it is well tolerated with only minor changes in the respective endpoints investigated. Recent systematic reviews of the clinical trials that assessed SMOF effects suggest that SMOF-based LEs may provide nutritional advantages over soybean-oil-based formulations, but there are currently too few studies for a consensus [17,43].

Another novel aspect of the present study is the characterization of apoptosis and necrosis in the alveolar compartment of the lungs in experimental ARDS, as well as the impact of LEs. Several studies have already shown that LEs per se are capable of inducing apoptosis [13,15,44]. Previously, our group used a murine model of LPS-induced ARDS to demonstrate that infusion of LCT augmented apoptosis in splenic and circulating lymphocytes and led to increased mortality [15]. Using the same model, olive-oil-based emulsions exhibited lower pro-apoptotic activity [15]. Similar results have also been reported using other experimental models, such as LPS-induced human monocyte leukemia-cell stimulation [45], ex-vivo analysis of human leukocytes after LCT infusion [14], and an in-vitro model of enterocyte apoptosis after incubation with various LEs [46]. In the present study, we observed that both LEs tested were capable of inducing both apoptosis and necrosis in alveolar immune cells under conditions of experimental ARDS. SMOF induced a higher percentage of apoptosis, especially in alveolar Mϕ-type cells, PMNs, and CD19+ lymphocytes, whereas animals that received LCT had higher levels of necrosis in the above-mentioned cell types. Whether these findings have any direct impact on the observed superior effects of SMOF compared to LCT in our murine ARDS model is unclear and needs further evaluation. Nevertheless, for the first time, these data indicate that (a) commonly used LEs for parenteral nutrition directly influence apoptosis and necrosis of cells in the alveolar compartment and (b) that significant differences exist between the two LEs tested concerning their pro-apoptotic and pro-necrotic properties. One limitation of our study is the use of the murine model of LPS-induced ARDS, which is evidently different from clinical and experimental ARDS caused by bacterial or viral infections. In spite of this aspect, the advantage of the model is its high degree of standardization and the formation of clear morphological changes typically observed in human ARDS, such as barrier dysfunction or the formation of pulmonary edema.

In summary, we have demonstrated that a modern, n-6-reduced LE mixture exerts anti-inflammatory and pro-resolving effects in the murine model of LPS-induced ARDS. Partial replacement of n-6 fatty acids with n-3/n-9 fatty acids may thus be of benefit for critically ill patients at risk for ARDS who require parenteral nutrition.

## Figures and Tables

**Table 1:** Fatty acid compositions of both lipid emulsions tested in the study.

Fatty Acid (g/100g)	LCT	SMOF
Medium chain: caprylic acid (C8:0)		10
Medium chain: capric acid (C10:0)		10
Medium chain: lauric acid (C12:0)		10
Long chain (LC) saturated: palmitic acid (C16:0)	10.4	5.9
LC saturated: stearic acid (C18:0)	4.4	1.8
LC mono-unsaturated: oleic acid (C18:1) n-9	22.5	25
LC poly-unsaturated: linoleic acid (C18:2) n-6	54.4	18.7
LC poly-unsaturated: α-Linolenic acid (C18:3) n-3	6.7	8.7
LC poly-unsaturated: eicosapentaenoic acid (C22:5) n-3		4.8
LC poly-unsaturated: docosahexaenoic acid (C22:6) n-3		3.5
Ratio n-6/n-3	8:1	1.3:1
